# A review on advances in chemical compound toxicology prediction

**DOI:** 10.3389/fbinf.2026.1974935

**Published:** 2026-10-12

**Authors:** Ahmed Hagag, Nada A. Elsayed, Ibrahim Abdelbaky

**Affiliations:** 1 Information Technology and Computing Program, Faculty of Computer Studies, Arab Open University, El-Shorouk, Egypt; 2 Department of Scientific Computing, Faculty of Computers and Artificial Intelligence, Benha University, Benha, Egypt; 3 Department of Artificial Intelligence, Faculty of Computers and Artificial Intelligence, Benha University, Benha, Egypt; 4 Computer Science Department, Faculty of Computers and Information Technology, Future University in Egypt, New Cairo, Egypt

**Keywords:** chemical toxicity, deep learning, explainable artificial intelligence, machine learning, molecular representations

## Abstract

The accurate prediction of chemical toxicity remains a major challenge in drug development and chemical safety assessment because conventional approaches can be costly, time-consuming, ethically constrained, and limited in predictive performance. This review examines how artificial intelligence (AI), particularly machine learning (ML) and deep learning (DL), is being applied to predictive toxicology. It covers conventional algorithms such as support vector machines (SVMs), decision trees (DTs), and ensemble methods, together with advanced approaches including graph neural networks (GNNs). The review evaluates AI-based methods across five organ-specific toxicity domains—hepatotoxicity, cardiotoxicity, respiratory toxicity, nephrotoxicity, and neurotoxicity—and critically compares molecular representations, data-integration strategies, model architectures, validation protocols, and interpretability approaches. Particular emphasis is placed on data quality, class imbalance, model explainability, applicability domains, external validation, and regulatory acceptance. By integrating recent methodological advances with persistent implementation challenges, this review provides a technical framework for developing more transparent, reliable, and generalizable computational tools for safety assessment in drug discovery and environmental health.

## 1 Introduction

The production and distribution of medicinal products represent significant advancements in healthcare, facilitating the prevention, diagnosis, and treatment of diseases that enhance both quality of life and life expectancy. However, this therapeutic potential carries inherent risks, as many chemical entities can cause adverse effects, ranging from discomfort to chronic organ damage and toxicity. This duality renders drug safety a paramount concern, demonstrating the value of accurate toxicological predictions in medication development (Zhang et al., 2025; Shehab et al., 2025a). Unrecognized toxicity can lead to drug-development failure and, after approval, may result in product withdrawal, substantial economic losses, and reduced public trust (Pérez Santín et al., 2021; Guo et al., 2023; Cavasotto and Scardino, 2022). Thorough safety assessments in the early stages of discovery can eliminate high-risk candidates or optimize candidates with unfavorable safety profiles, thereby increasing the likelihood of success in research and development (R&D) (Zhang et al., 2025). Addressing the need for predictive accuracy requires robust toxicological assessments that account for the various ways chemicals can affect individuals. These assessments are typically based on the duration of exposure (acute or chronic) or the biological targets affected, such as carcinogenicity, genotoxicity, and organ-specific toxicity. Traditionally, standard safety assessments rely on *in vitro* assays and animal testing techniques (Shehab et al., 2025a; Pérez Santín et al., 2021). While these methods yield valuable information, they are increasingly viewed as less effective due to their high resource demands, lengthy timelines, and ethical concerns regarding the use of animals (Guo et al., 2023). Additionally, interspecies physiological differences often limit the translational validity of animal models, as evidenced by instances where drugs exhibited significant human toxicity that was not identified in preclinical animal studies. A comprehensive overview of the artificial intelligence (AI)-based toxicity prediction pipeline is illustrated in Figure 1. To overcome these challenges, integrating extensive toxicology data with advances in computer technology has led to a transformative shift in safety evaluation. AI techniques, particularly machine learning (ML) and deep learning (DL), have advanced toxicity prediction by identifying complex nonlinear patterns in high-dimensional chemical and biological datasets (Wu and Wang, 2018; Miotto et al., 2018). While quantitative structure-activity relationship (QSAR) modelling, which connects chemical structure to biological activity, has long served as a computational foundation, traditional approaches have often struggled with the diversity of chemical datasets. In contrast, modern ML and DL frameworks can effectively extract key molecular features, thereby enhancing the accuracy of predictions regarding toxicological outcomes. Access to extensive toxicity databases and powerful computing resources supports this capability (Guo et al., 2023; Cavasotto and Scardino, 2022). This computational shift enables rapid, cost-effective screening of large compound libraries during the early discovery phase, thereby mitigating risks in development pipelines and resulting in significant cost savings (Mullowney et al., 2023). AI models can also be updated as new data become available, enabling iterative refinement of predictive frameworks (Zhang et al., 2025). Considering these advances, this review critically examines AI-based approaches for chemical toxicity prediction, with emphasis on toxicity data resources, molecular representations, ML and DL models, validation, interpretability, and major organ-specific toxicity endpoints. Particular attention is given to methodological limitations, model reliability, and challenges affecting generalizability and practical application.

**FIGURE 1:**
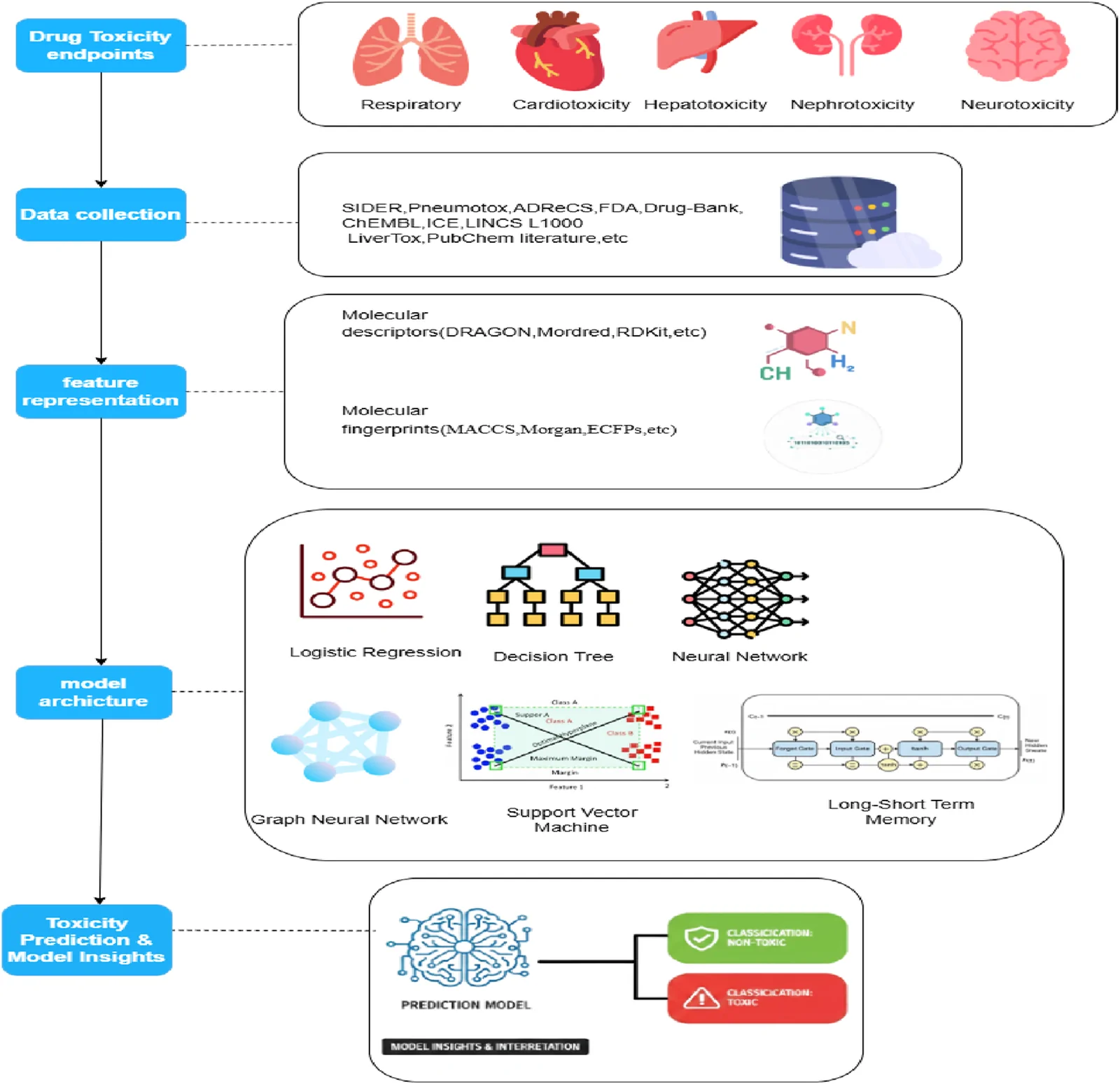
Presents a schematic workflow for computational drug-toxicity prediction. The process includes: (i) defining toxicity endpoints (e.g., cardiotoxicity, hepatotoxicity, and neurotoxicity); (ii) acquiring data from publicly accessible databases; (iii) representing molecules using descriptors, fingerprints, or graph-based features; (iv) developing models using ML and DL methods (e.g., SVMs, neural networks, and graph-based models); and (v) generating toxicity predictions together with model interpretation.

The remainder of this paper is organized as follows. Section 2 describes the research methodology and review protocol. Section 3 summarizes commonly used toxicity datasets and critically compares their strengths and limitations. Section 4 discusses molecular representation, ML and DL model architectures, model evaluation and validation, explainability, applicability domains, and regulatory considerations. Section 5 reviews organ-specific toxicity-prediction studies and provides a cross-organ comparative synthesis. Section 6 discusses limitations and future research directions, and Section 7 presents the conclusion.

## 2 Research methodology and review protocol

### 2.1 Review scope

This review summarizes recent advances in AI, ML, and DL for predicting chemical- and drug-induced toxicity, with particular emphasis on five major organ-specific domains: respiratory toxicity, cardiotoxicity, nephrotoxicity, hepatotoxicity, and neurotoxicity. The review covers conventional ML, DL, graph-based, ensemble, and multimodal approaches using diverse molecular representations. In addition to predictive performance, the selected studies are critically assessed in terms of dataset size and composition, class distribution, molecular representation, data-splitting and validation strategies, independent external validation, model interpretability, applicability domain, and major methodological limitations.

### 2.2 Search strategy and eligibility criteria

A structured literature search was conducted to identify studies applying AI, ML, and DL to chemical and drug toxicity prediction. Searches were performed primarily in Google Scholar and ScienceDirect and were supplemented by other academic databases, publisher platforms, and relevant scientific resources to broaden coverage. The search strategy combined terms related to toxicity prediction, computational methods, molecular representation, and organ-specific toxicity endpoints. Search terms included “chemical toxicity prediction,” “drug toxicity prediction,” “artificial intelligence,” “machine learning,” “deep learning,” “QSAR,” “molecular descriptors,” “molecular fingerprints,” “graph neural network,” “explainable artificial intelligence,” “respiratory toxicity,” “cardiotoxicity,” “nephrotoxicity,” “hepatotoxicity,” “drug-induced liver injury,” and “neurotoxicity.” These terms were used individually and in combination. Additional records were identified through related-article searches and other scholarly sources. The strategy was designed to provide broad coverage of recent peer-reviewed literature rather than restrict retrieval to a single bibliographic database. For the organ-specific comparative analysis, the literature search primarily focused on peer-reviewed original research articles published from 2021 through 2026. Studies were selected using predefined eligibility criteria to ensure relevance and sufficient methodological information for comparison. Studies were included if they: (1) investigated toxicity associated with chemicals, drugs, or small molecules; (2) applied AI, ML, DL, QSAR, graph-based learning, or another computational modeling approach; (3) addressed at least one of the five organ-specific toxicity domains considered in this review; (4) reported sufficient information on the dataset, input features or molecular representation, predictive model, validation strategy, and performance evaluation; and (5) were published as peer-reviewed original research articles. Studies were excluded from the comparative organ-specific tables if they: (1) were not directly related to chemical or drug toxicity prediction; (2) focused exclusively on disease diagnosis, medical-image segmentation, or other clinical prediction tasks unrelated to chemical toxicity; (3) provided insufficient methodological or evaluation details for comparison; (4) duplicated another publication or analysis of the same study; or (5) were reviews, editorials, conference abstracts, or other non-primary research articles.

### 2.3 Study selection and data extraction

Identified records were initially screened by title and abstract for relevance to chemical or drug toxicity prediction and for the availability of sufficient methodological and performance information. Full texts of potentially eligible studies were then assessed against the predefined inclusion and exclusion criteria. Following screening and eligibility assessment, 40 primary organ-specific studies published from 2021 through 2026 were included in the comparative synthesis. Their distribution across the five organ-specific toxicity domains is presented in Table 1. By publication year, the included studies comprised 2 articles from 2021, 6 from 2022, 1 from 2023, 8 from 2024, 18 from 2025, to 5 from 2026. For each included organ-specific study, data were extracted on the toxicity endpoint, dataset and sample size, positive and negative class distribution when reported, molecular representation or input features, predictive model, data-splitting and validation strategy, reported performance metrics, independent external validation, and major methodological limitations. These characteristics were used to construct the organ-specific comparative tables and to support the critical synthesis presented in this review.

**TABLE 1:** Distribution of studies included in the organ-specific comparative synthesis.

Toxicity domain	Number of studies
Respiratory toxicity	7
Cardiotoxicity	10
Nephrotoxicity	7
Hepatotoxicity	10
Neurotoxicity	6
Total	40

## 3 Toxicity data resources

Datasets are fundamental to the development and validation of toxicity-prediction models and may include chemical structures, biological-assay results, molecular features, adverse-event information, and toxicity endpoints. Because toxicity databases differ in size, source, biological context, label quality, and endpoint definition, their suitability for computational modeling varies considerably. This section summarizes several commonly used resources and discusses their relevance to computational toxicity prediction.

### 3.1 Major toxicity databases

1. Side Effect Resource (SIDER):

SIDER links marketed drugs to reported adverse drug reactions (ADRs) and is particularly useful for pharmacovigilance and drug-side-effect modeling, although it primarily reflects clinical safety information rather than standardized experimental toxicity measurements (Kuhn et al., 2016).

2. Food and Drug Administration (FDA) Adverse Event Reporting System (FAERS):

FAERS contains spontaneous post-marketing adverse event reports and supports pharmacovigilance and safety-signal detection. Its use in predictive modeling is limited by reporting bias, duplicate records, missing information, and uncertain negative examples (Liu H. et al., 2025).

3. Adverse Drug Reaction Classification System (ADReCS):

ADReCS provides a standardized hierarchical ontology for ADR terminology and links adverse reactions to drug-related information. It supports harmonization of adverse-event labels but may inherit limitations from the underlying clinical and pharmacovigilance sources (Cai et al., 2015).

4. Pneumotox:

Pneumotox is an organ-specific clinical resource for drug- and chemical-induced respiratory disorders. Its curated clinical information is valuable for respiratory safety assessment, although it is not directly structured as a quantitative QSAR dataset (Camus et al., 2014).

5. PubChem:

PubChem integrates chemical structures, physicochemical properties, bioassay results, and toxicity-related information across broad chemical space. However, heterogeneous assays and endpoint definitions often require substantial preprocessing and harmonization (Kim et al., 2016).

6. DrugBank:

DrugBank integrates chemical, pharmacological, target, interaction, indication, and safety information for approved and investigational drugs. Its drug-centered coverage is useful for ADMET modeling but may limit generalization to environmental or industrial chemicals (Wishart et al., 2007).

7. ChEMBL:

ChEMBL provides curated chemical structures, targets, bioactivity measurements, and ADMET-related information. However, heterogeneity in assays and endpoint definitions requires careful curation (Davies et al., 2015).

8. Integrated Chemical Environment (ICE):

ICE integrates *in vivo*, *in vitro*, and *in silico* toxicity data from multiple sources, including ToxCast and Tox21, and supports integrated and non-animal safety assessment approaches (Daniel et al., 2022).

9. Integrated Network-based Cellular Signatures (LINCS) L1000:

LINCS L1000 provides transcriptomic responses of human cells exposed to chemical and genetic perturbations and can support mechanistic and multimodal toxicity modeling, although coverage and experimental conditions vary across compounds (Subramanian et al., 2017).

10. LiverTox:

LiverTox summarizes clinical evidence on drug-induced liver injury (DILI) and is highly relevant to hepatotoxicity, although its narrative clinical evidence requires careful curation before use as ML labels (Injury and LiverTox, 2012).

### 3.2 Toxicity data types

The data sources used in computational toxicology are heterogeneous and represent different levels of biological evidence; therefore, they should not be treated as interchangeable. Chemical toxicity prediction generally aims to estimate the harmful effects of chemical compounds using structural, experimental, or biological information. In contrast, adverse drug reaction (ADR) prediction focuses on undesirable effects observed following drug exposure in humans. Accordingly, databases such as SIDER, FAERS, and ADReCS primarily contain drug-related adverse-event or clinical safety information rather than standardized experimental measurements of intrinsic chemical toxicity (Kuhn et al., 2016; Liu H. et al., 2025; Cai et al., 2015).

Experimental toxicity data also differ according to the biological system used for toxicity assessment. Traditional testing relies on *in vivo* animal studies, whereas modern high-throughput toxicology increasingly uses *in vitro* cell-based and biochemical assays (Lynch et al., 2024). These endpoints represent different levels of biological evidence and should not be considered interchangeable, particularly because species differences and experimental context can limit extrapolation from *in vitro* or animal findings to human toxicity (Clewell and Fuchsman, 2024; Lin, 1998).

### 3.3 Comparison of toxicity data sources

Toxicity databases differ substantially in data sources, biological relevance, chemical-space coverage, and endpoint definitions. These differences directly affect their suitability for ML applications. Broad chemical and experimental resources such as PubChem, ChEMBL, and ICE (Kim et al., 2016; Davies et al., 2015; Daniel et al., 2022) differ fundamentally from clinical adverse-event sources such as SIDER and FAERS (Kuhn et al., 2016; Liu H. et al., 2025), transcriptomic resources such as LINCS L1000 (Subramanian et al., 2017), and organ-specific resources such as Pneumotox and LiverTox (Camus et al., 2014; Injury and LiverTox, 2012).

These differences can affect model reliability and generalizability, particularly when datasets vary in assay conditions, species, exposure duration, endpoint definitions, label quality, or chemical-space coverage. Class imbalance and uncertain class definitions may further influence model training and evaluation. Therefore, careful dataset construction, molecular standardization, endpoint harmonization, and duplicate removal are important before model development (Guo et al., 2023; Cavasotto and Scardino, 2022; Seal et al., 2025). Table 2 summarizes the main toxicity datasets discussed in this review. Database selection should ultimately reflect the target endpoint and intended application. Label reliability, chemical and biological coverage, class distribution, data quality, and the availability of independent validation data should be considered. Validation using chemically diverse external compounds provides stronger evidence of model generalizability (Seal et al., 2025).

**TABLE 2:** Critical comparison of commonly used toxicity data sources.

Database	Main data/Coverage	Key strengths	Main limitations/ML concerns	Typical suitability
SIDER (Kuhn et al., 2016)	Clinical ADRs from marketed drugs	Clinically relevant human ADR information	Label heterogeneity; marketed drugs only; uncertain negatives	Drug adverse-event and pharmacovigilance modeling
FAERS (Liu et al., 2025a)	Post-marketing adverse-event reports	Large real-world source for safety-signal detection	Reporting bias, duplicates, missing data, uncertain negatives	Pharmacovigilance and adverse-event prediction
ADReCS (Cai et al., 2015)	Standardized ADR ontology and drug information	Standardized hierarchical ADR terminology	Dependent on underlying clinical/ADR sources	ADR classification and drug-safety modeling
Pneumotox (Camus et al., 2014)	Drug- and chemical-induced respiratory disorders	Focused respiratory safety information	Organ-specific, largely narrative data requiring curation	Respiratory toxicity assessment
PubChem (Kim et al., 2016)	Chemical structures, bioassays, and toxicity data	Broad chemical and assay coverage	Heterogeneous assays and endpoints	Chemical toxicity and QSAR modeling
DrugBank (Wishart et al., 2007)	Drug, target, interaction, and ADMET data	Rich pharmacological and drug-target information	Drug-centered chemical space	Drug-safety and ADMET modeling
ChEMBL (Davies et al., 2015)	Curated chemical and bioactivity data	Curated bioactivity and assay information	Assay and endpoint heterogeneity	Bioactivity and toxicity modeling
ICE (Daniel et al., 2022)	Integrated *in vivo*, *in vitro*, and *in silico* toxicity data	Integration of multiple toxicity evidence types	Heterogeneous sources require harmonization	Chemical-safety and multimodal modeling
LINCS L1000 (Subramanian et al., 2017)	Transcriptomic responses to chemical exposure	Mechanistic transcriptomic information	High dimensionality, variable coverage, preprocessing needs	Mechanistic and multimodal modeling
LiverTox (Injury and LiverTox, 2012)	Clinical DILI information	Clinically relevant DILI evidence	Liver-specific, narrative evidence, complex label construction	Hepatotoxicity and DILI prediction

## 4 Computational toxicity modeling

The performance of computational toxicity-prediction models depends strongly on molecular representation, model architecture, data quality, and validation strategy. This section examines commonly used molecular representations and ML/DL approaches, together with model evaluation, explainability, applicability-domain assessment, prediction reliability, and regulatory considerations.

### 4.1 Molecular representation

Molecular representation determines how chemical information is encoded for computational modeling. Common representations include molecular descriptors, structural fingerprints, Simplified Molecular Input Line Entry System strings (SMILES), molecular graphs, and learned molecular embeddings. The choice of representation can substantially influence model performance because different representations capture different structural, physicochemical, and contextual information.

#### 4.1.1 Molecular fingerprints

Molecular fingerprints encode molecular structure and properties as bit strings or numerical vectors. Each bit signifies the presence or absence of a specific chemical property or substructure (Deng et al., 2025). There are various types available, including.

1. MACCS:

MACCS fingerprints encode molecules using 166 predefined structural keys representing characteristic substructures (Kuwahara and Gao, 2021). An example of a MACCS fingerprint representation is illustrated in Figure 2.

2. Extended Connectivity Fingerprints (ECFPs):

**FIGURE 2:**
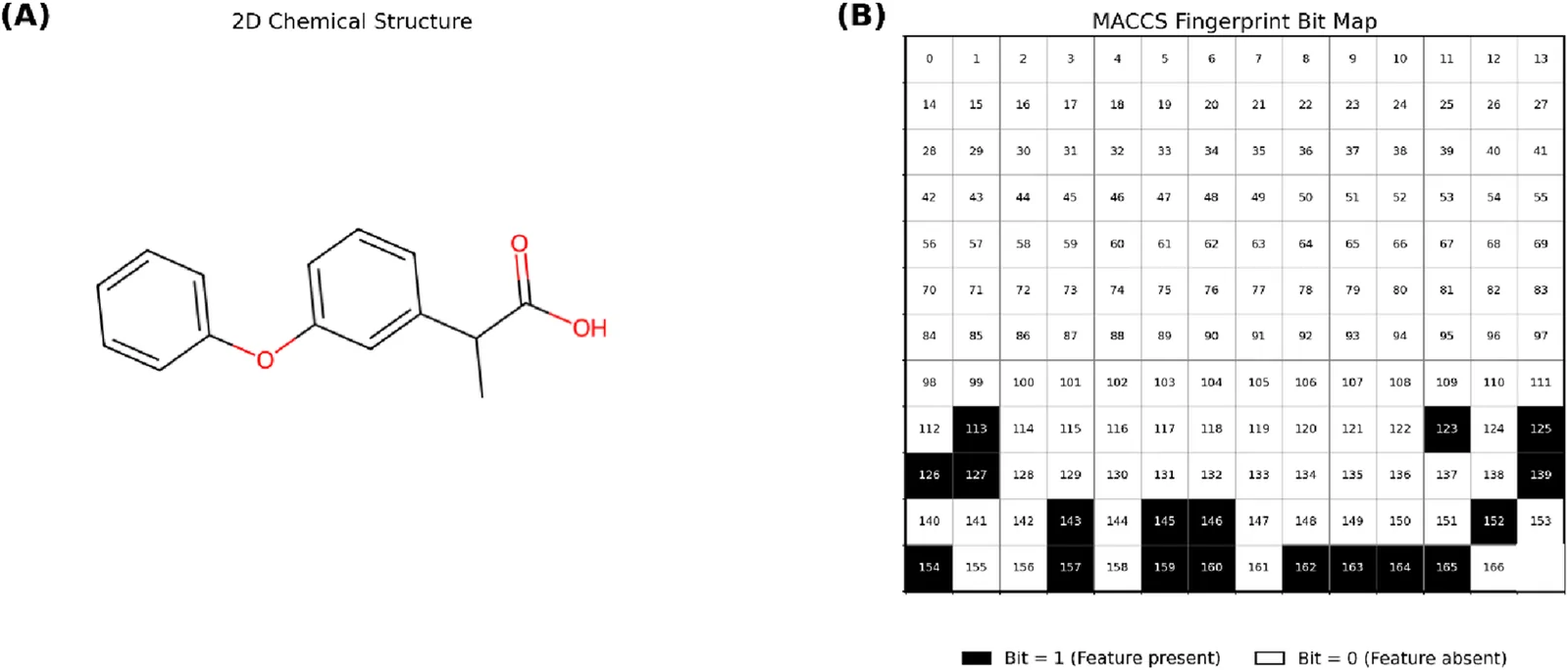
Example of a MACCS fingerprint for fenoprofen, showing **(A)** the molecular structure and **(B)** the corresponding 166-key binary representation.

ECFPs encode circular atomic neighborhoods and are widely used for molecular similarity, structure–activity modeling, and ML applications (Rogers and Hahn, 2010). An ECFP representation is illustrated in Figure 3.

3. The Chemistry Development Kit (CDK):

**FIGURE 3:**
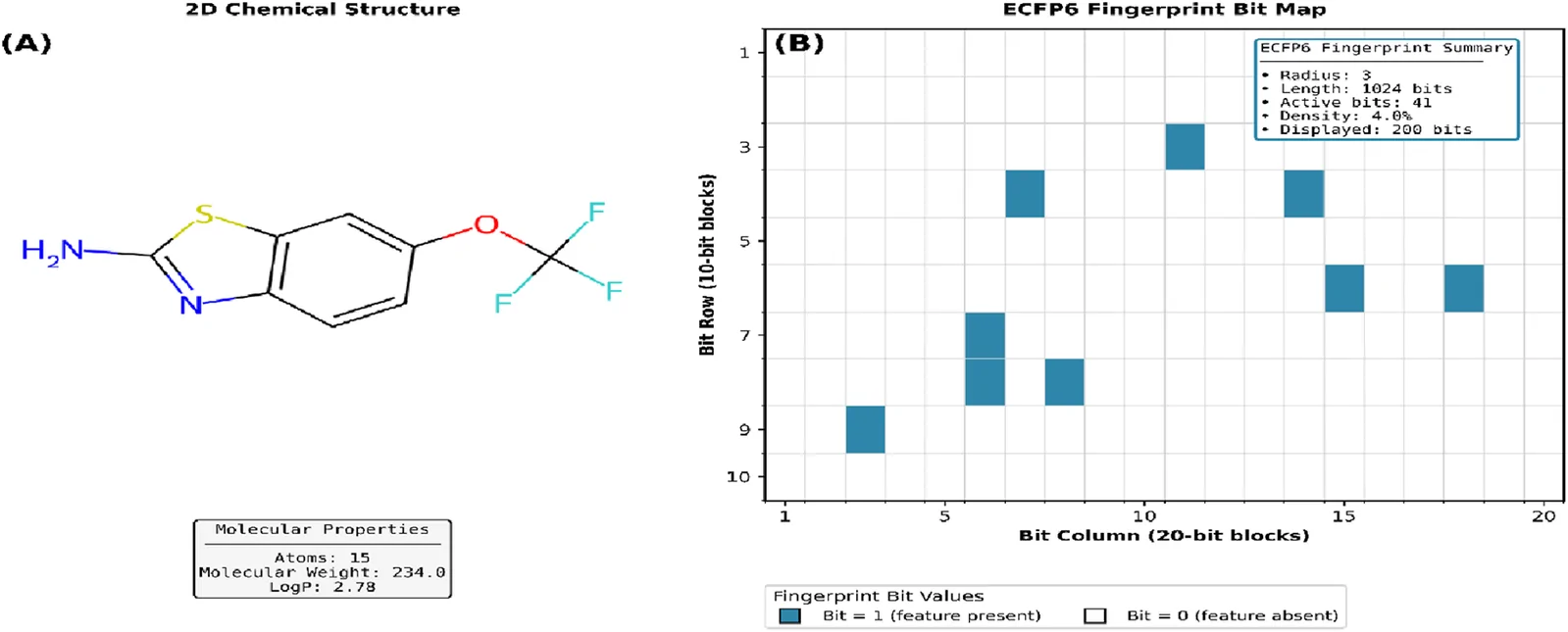
Example of an ECFP6 representation for riluzole, showing **(A)** the molecular structure and **(B)** the corresponding binary fingerprint.

CDK provides several fingerprint types, including substructure-, circular-, ring-, and electrotopological-state-based representations, supporting diverse cheminformatics applications (Willighagen et al., 2017).

4. Graph-based representations:

Graph-based representations encode atoms as nodes and bonds as edges, preserving molecular connectivity and enabling direct learning from molecular structure, particularly with graph neural networks (GNNs) (Nguyen-Vo et al., 2024). An illustrative example of graph-based representations appears in Figure 4.

**FIGURE 4:**
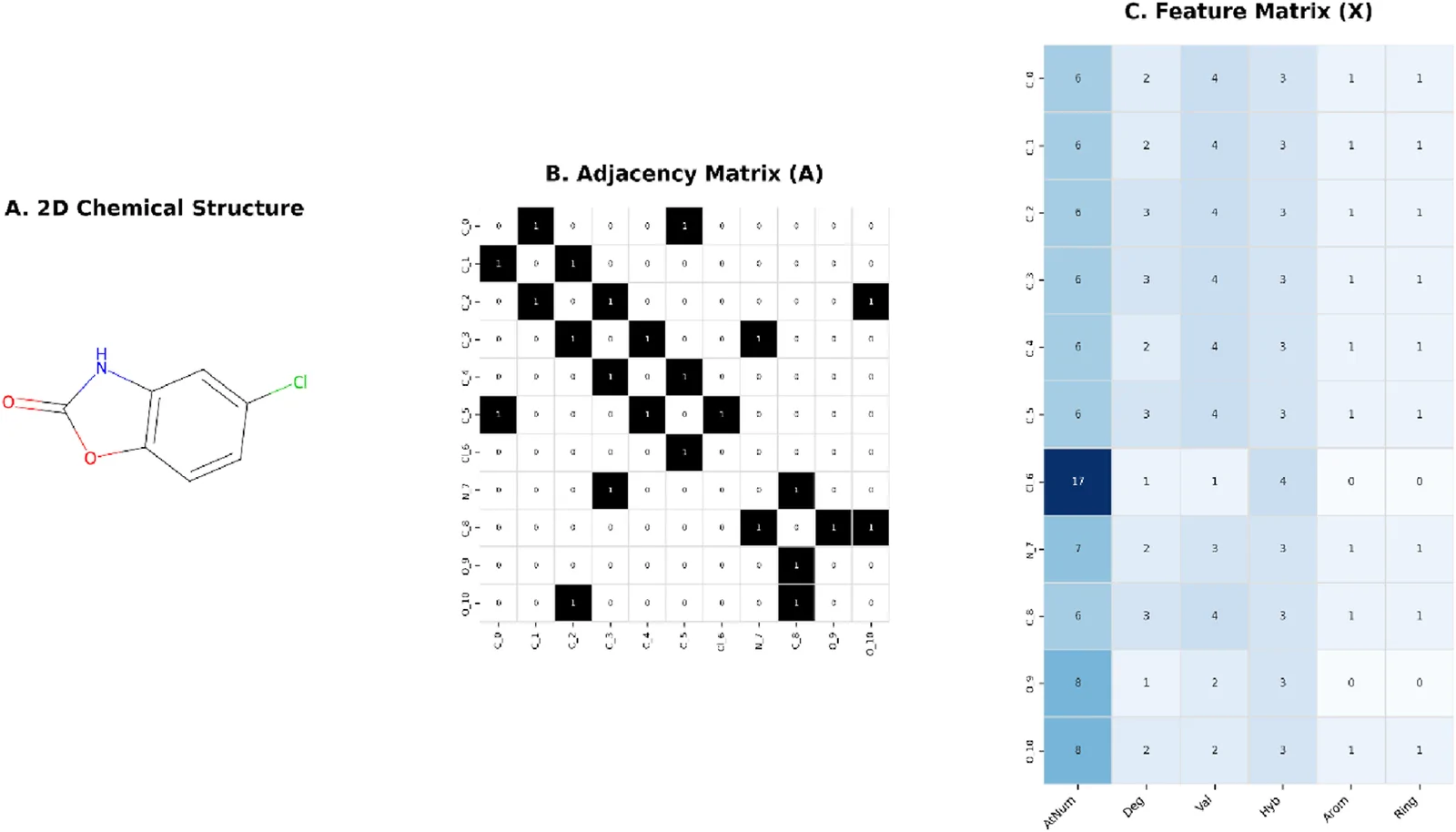
Example of a molecular graph representation showing **(A)** the molecular structure, **(B)** the adjacency matrix, and **(C)** the node-feature matrix.

#### 4.1.2 Molecular descriptors

Molecular descriptors are numerical values that characterize structural, physicochemical, and topological properties of molecules and are widely used in computational chemistry and toxicity prediction. Various tools are available for calculating these descriptors, including.

1. DRAGON:

DRAGON provides a large collection of constitutional, topological, geometric, and physicochemical molecular descriptors and is widely used in QSAR and cheminformatics applications (Tetko et al., 2005).

2. Mordred:

Mordred is an open-source tool that calculates more than 1,800 two- and three-dimensional molecular descriptors for cheminformatics and structure–activity modeling (Moriwaki et al., 2018).

A visualization of the Mordred descriptors representation is included in Figure 5.

3. RDKit:

**FIGURE 5:**
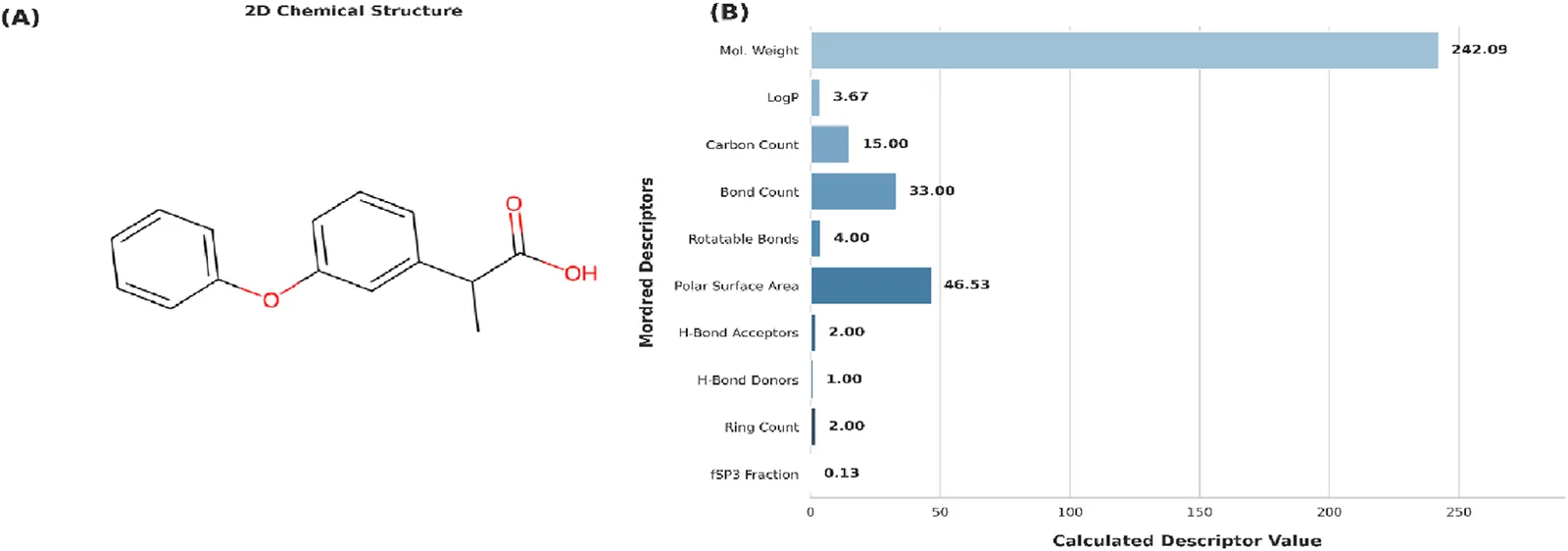
Example of molecular descriptor representation for fenoprofen using Mordred, showing **(A)** the two-dimensional molecular structure and **(B)** selected calculated molecular descriptors.

RDKit is an open-source cheminformatics toolkit that computes a broad range of molecular descriptors, including physicochemical, topological, and structural features (Landrum, 2013). An illustrative example of RDKit descriptors appears in Figure 6.

4. PaDEL:

**FIGURE 6:**
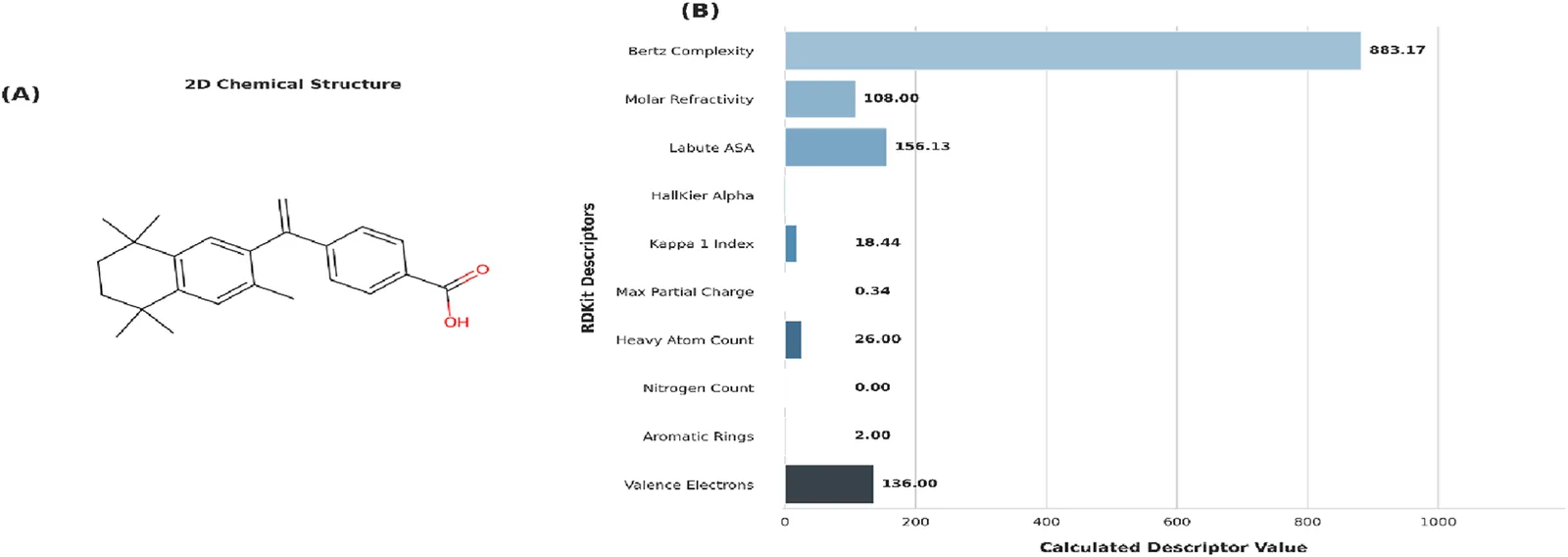
Example of RDKit molecular descriptors for bexarotene, showing **(A)** the two-dimensional molecular structure and **(B)** selected calculated descriptors.

PaDEL-Descriptor is an open-source software package that calculates hundreds of one-, two-, and three-dimensional molecular descriptors together with multiple fingerprint types (Yap, 2011).

5. Mold2:

Mold2 is an FDA-developed tool that generates hundreds of two-dimensional molecular descriptors and has been applied in computational toxicology and QSAR modeling (Hong et al., 2008).

### 4.2 ML and DL models

ML methods remain widely used in toxicity prediction because they can model relationships between molecular features and toxicological outcomes. Common supervised-learning algorithms include RF (Breiman, 2001), SVM (Guido et al., 2024), LR (Dey et al., 2025), decision tree (DT) (Blockeel et al., 2023), k-nearest neighbors (KNN) (Suyal and Goyal, 2022), naive Bayes (NB) (Pajila et al., 2023), XGBoost (Bentéjac et al., 2021), and LightGBM (Anggreainy, 2024). Furthermore, DL methods provide an alternative approach for learning complex patterns from chemical and biological data. Common architectures include deep neural networks (DNNs), convolutional neural networks (CNNs), GNNs, and transformer-based models. DNNs can learn nonlinear relationships from molecular descriptors and fingerprints, whereas CNNs can extract hierarchical features from molecular images or other structured representations. GNNs can learn directly from molecular graph representations (Miao et al., 2023), while transformer-based models can learn contextual molecular representations from SMILES strings or pre-trained molecular embeddings (Mswahili and Jeong, 2024; Bai et al., 2019). Chemical-specific factors are also important determinants of model performance in toxicological applications. Chirality, protonation and tautomeric states, and 3D conformational sampling can influence molecular representation and biological activity. In addition, learned molecular embeddings and pre-trained chemical language models, such as ChemBERTa, SMILES-BERT, MolBERT, and MoLFormer, can provide richer molecular representations for toxicity prediction. Accordingly, the suitability of ML and DL approaches depends on the available data, toxicity endpoint, molecular representation, and preprocessing strategy rather than on model complexity alone.

### 4.3 Model evaluation and comparison

ML and DL models have achieved strong performance in toxicity prediction; however, cross-study comparisons should be interpreted cautiously. Differences in ACC, ROC-AUC, and other metrics may reflect variation in dataset composition, toxicity endpoints, class distribution, molecular representation, and validation strategy rather than intrinsic superiority of one algorithm. Conventional ML methods such as RF, SVM, XGBoost, and LightGBM remain competitive for small-to medium-sized datasets with informative descriptors or fingerprints, whereas GNNs, transformers, and multimodal DL approaches may benefit from larger datasets and richer representations. No single architecture consistently performs best across toxicity-prediction tasks.

Model performance is strongly influenced by molecular representation and validation design. Fingerprints and descriptors encode predefined structural and physicochemical information, whereas graph-based and learned representations can capture more complex molecular relationships. Random train/test splitting may yield optimistic results when structurally similar compounds or close analogues occur in both sets, while scaffold-based, cluster-based, temporal, and independent external validation provide more rigorous assessments of generalization to novel chemical space (Guo et al., 2023; Seal et al., 2025).

Data leakage can further inflate reported performance when information from the full dataset is used during preprocessing, feature selection, normalization, or hyperparameter optimization before the final train/test separation. Independent external validation using compounds from a separate source provides particularly strong evidence of model generalizability, and results obtained under different splitting or validation strategies should therefore not be compared directly.

Class imbalance is another important challenge because toxic compounds may be less frequent than non-toxic compounds. Strategies such as class weighting, oversampling, undersampling, SMOTE, focal loss, threshold optimization, and data augmentation can reduce imbalance-related bias (Masood et al., 2025). SMOTE should be applied cautiously because synthetic feature-space samples may not preserve structural validity or biological relevance (Correia et al., 2024), while SMILES-based augmentation should preserve molecular identity and toxicity labels (Cutropia et al., 2026).

Accuracy (ACC) alone is insufficient for evaluating toxicity-prediction models, particularly for imbalanced datasets. Evaluation should therefore include complementary metrics such as sensitivity, specificity, precision, F1-score, balanced accuracy (BA), Matthews correlation coefficient (MCC), ROC-AUC, and PR-AUC. PR-AUC is often more informative when toxic compounds are rare, while calibration should also be considered when predicted probabilities are intended to support risk assessment (Saito and Rehmsmeier, 2015).

### 4.4 Explainability and mechanistic interpretation

Explainable artificial intelligence (XAI) methods can improve model transparency by identifying molecular features that contribute to toxicity predictions. Common techniques include SHapley Additive exPlanations (SHAP), feature-importance analysis, attention mechanisms, saliency methods, and structural-alert identification. However, model interpretability should be distinguished from biological or mechanistic validation. High SHAP values, attention weights, or atom-level attribution maps may identify features associated with a prediction but do not establish that those features cause the observed toxicological effect. Attribution results may also vary with molecular representation, correlated descriptors, model architecture, and explanation method. Therefore, XAI outputs should be interpreted as explanations of model behavior or hypotheses rather than direct evidence of toxicological causality. Mechanistic interpretation requires additional support from experimental validation, target-interaction data, toxicogenomics, pathway analysis, adverse outcome pathways, or other independent biological evidence (Kabdraisova et al., 2026; Lavecchia, 2025).

### 4.5 Applicability, reliability, and regulatory considerations

The applicability domain (AD) defines the region of chemical space in which a toxicity-prediction model is expected to provide reliable predictions. Predictions for compounds that differ substantially from the training data may be unreliable even when the model shows high internal performance. ADs can be assessed using several approaches, including structural similarity, molecular-descriptor distances, nearest-neighbor methods, leverage-based approaches, clustering, and other chemical-space measures. Predictions outside, or near the boundaries of, the AD should be interpreted cautiously, and uncertainty assessment can provide complementary information on prediction reliability (Seal et al., 2025; OECD, 2024).

Strong retrospective performance alone is insufficient to establish suitability for regulatory use. Regulatory confidence depends on predictive performance, independent external validation, clearly defined endpoints, transparent and reproducible workflows, applicability-domain assessment, uncertainty evaluation, and adequate model documentation. Documentation should describe the input data, molecular representation, algorithm, preprocessing, model parameters, validation procedure, and known limitations. Regulatory acceptance should therefore consider performance, external validation, AD, uncertainty, reproducibility, transparency, and biological plausibility rather than high ACC or ROC-AUC values alone (OECD, 2024; Gissi et al., 2024).

## 5 Organ toxicity

Organ-specific toxicity is a critical area of toxicological research because chemical compounds can cause various adverse effects in organs and physiological systems. Computational models have been significantly developed to help predict and assess these effects. This section presents a brief review of recent studies on respiratory toxicity, cardiotoxicity, nephrotoxicity, hepatotoxicity, and neurotoxicity.

### 5.1 Respiratory toxicity

Wang et al. (Wang et al., 2021) developed QSAR models that predict respiratory toxicity with an ACC of 93.3% using RF with PubChem fingerprints. They utilized structural alerts and applicability domain (AD) analysis to enhance model reliability; however, the chemical applicability domain was limited, and the external validation set was structurally similar to the training set and therefore not fully representative. Consequently, Jaganathan et al. (Jaganathan et al., 2022) developed an interpretable SVM model to predict respiratory toxicity, achieving an ACC of 86.2% using hybrid feature selection. The model employed SHAP analysis to identify critical molecular descriptors associated with toxicity; however, this *post hoc* method does not establish biological causality. Significant limitations include the exclusion of metals and inorganic compounds, as well as potential bias in the construction of the nontoxic class. In another effort, Jaganathan et al. (Jaganathan et al., 2025) developed a LightGBM model to predict chemical lung toxicity, achieving an ACC of 86.88% and a ROC-AUC of 0.931. They used SHAP analysis to improve interpretability and identify chemical descriptors that contributed to toxicity prediction. However, a major limitation of their study is its reliance on 2D molecular models and *post hoc* interpretation methods. Furthermore, Shehab et al. (Shehab et al., 2025b) created an ML framework for predicting respiratory toxicity by integrating molecular descriptors with SMILES features. Their RF model achieved an ACC of 92.2% and a ROC-AUC of 0.970 during external validation and incorporated an AD assessment. To address class imbalance, they employed SMOTE and optimized hyperparameters using OPTUNA. Nonetheless, one limitation of the study is the model’s interpretability. Meanwhile, Kelleci Çelik and Sarıaltın (2025) developed a QSAR model to predict drug-induced interstitial pneumonitis, a serious type of lung toxicity. This model employs an LR classifier, achieving an ACC of 95.72%. They used SHAP analysis to obtain global and local explanations that may support molecular-design decisions. However, the model is restricted to drug-like molecules and is not designed for salts or inorganic compounds. Additionally, Li et al. (Li et al., 2025) developed DL models to predict respiratory toxicity endpoints, such as pulmonary embolism and pulmonary arterial hypertension, achieving a ROC-AUC of 0.97. Their methodology integrated structural alerts and SHAP analysis to enhance the models’ interpretability. Despite this, the study faces limitations, including challenges in selecting negative data and the potential for specificity bias. In contrast, Chushak et al. (Chushak et al., 2025) demonstrated progress by integrating structural alerts with ML models to develop a consensus model for predicting respiratory irritation and sensitization, with ACC values of 88% and 86% for respiratory irritation and sensitization, respectively. The study also identified distinct structural alerts that may support mechanistic interpretation. However, the limited dataset and the inclusion of alternative negative data restrict its generalizability.

Table 3 summarizes the reviewed studies on respiratory-toxicity prediction. Recent work has used diverse ML and DL approaches, with reported ACC values of approximately 86%–97%. Across these studies, recurrent challenges include limited chemical-domain coverage, uncertainty in negative-class construction, class imbalance, and incomplete external validation.

**TABLE 3:** Overview of previous research efforts in respiratory-toxicity prediction.

Study	Dataset(s)/sample size (N)	Endpoint	Positive/Negative	Features	Training model(s)	Split/validation	Best performance	Independent validation	Major limitation(s)
Wang et al. (2021)	Pneumotox, ADReCS, HCIS/literature; N = 2,529	Binary respiratory toxicity	Train: 1,043/826 Test: 261/206 Validation: 136/57	PubChem, MACCS, E-state, Klekota–Roth and other fingerprints	DT, SVM, RF, NB, ANN, KNN	80/20 + 10-fold CV + external set	RF/PubChem fingerprint external (ACC 0.933, ROC-AUC 0.936)	Yes	Limited chemical; applicability domain
Jaganathan et al. (2022)	Wang et al., SIDER, IntSide; 1,869 train; 465 test; 193 external	Chemical respiratory toxicity	Train: 1,043/826 Test: 259/206 Validation: 136/57	PaDEL descriptors	SVM, MLP, XGBoost, RF, LR, KNN, NB, ABDT	80/20 + 10-fold CV + external set	SVM (ACC 0.86 ROC-AUC 0.91)	Yes	Metals/inorganics excluded
Jaganathan et al. (2025)	Wang et al., 1,869 train; 465 test; 193 external	Pulmonary toxicity	Train 1,043/826; Test 259/206 Validation: 136/57	Morgan fingerprints, RDKit, Mordred	RF, Extra Classifier, AdaBoost, GB, XGBoost, LightGBM, CatBoost	10-fold CV + external sets	LightGBM (ACC 0.86 ROC-AUC 0.93, MCC 0.73)	Yes	2D representation; *post hoc* interpretation Limited dataset size
Shehab et al. (2025a)	Pneumotox, SIDER HCIS, ADReCS	Respiratory toxicity	Train: 1,043/826 Test: 261/206 Validation: 136/57	RDKit descriptors, TF-IDF SMILES	RF, SVC, LR, MLP, AdaBoost, GB	Internal + external validation	RF: external (ACC 0.922, ROC-AUC 0.970)	Yes	Limited interpretability; synthetic oversampling (SMOTE)
Kelleci Çelik and Sarıaltın (2025)	Pneumotox, SIDER 4.5, FDA; N = 468 APIs	Drug-induced interstitial pneumonitis	Train: 207/167; Test: 48/46	PaDEL descriptors	KStar, NB, LR, IBK	80/20 + 10-fold CV + external test	LR (ACC 0.957 internal; 0.947 external)	Yes	Restricted to drug-like molecules
Li et al. (2025)	SIDER, PNEUMOTOX; endpoint-specific	Eight respiratory toxicity endpoints	Train: 869/879 Validation: 203/235	MACCS fingerprints	fully connected DNN	80/20 + 10-fold CV + external set	ROC-AUC 0.97, ACC 0.95	Yes	Negative-label selection and specificity bias
Chushak et al. (2025)	ECHA, NITE, HCIS, OECD, literature; 1,251 irritation; 419 sensitization	Respiratory irritation and sensitization	624/627 (irritation); 208/211 (sensitization)	Descriptors, fragments, graph representations	ASNN, RF, KGNN, Transformer/CNN, consensus	5-fold CV + 80/20	Consensus: (irritation ACC 0.88; sensitization ACC 0.86)	No	Small/heterogeneous datasets; negative-data limitations

### 5.2 Cardiotoxicity

Ye et al. (Ye et al., 2022) developed ML models to predict DILI and cardiotoxicity (DICT). Their findings indicated that models relying solely on chemical structures outperformed those incorporating Tox21 assay data, achieving a DICT ROC-AUC of 0.83 with NB. However, a trade-off was evident: although structure-based models demonstrated higher predictive performance, they provided less direct mechanistic insight than the less predictive *in vitro* assay data, which were more informative for elucidating the biological pathways and mechanisms underlying toxicity. This highlights an important challenge in early safety assessment: balancing predictive performance with biological interpretability. Similarly, Delre et al. (Delre et al., 2022) developed consensus QSAR models intended to predict hERG cardiotoxicity. A key feature of these models is their strict AD, which improves reliability. While this feature increased balanced accuracy (BA) to 0.91 with SVM and RF, it also reduced chemical coverage, resulting in lower performance during temporal validation. Additionally, excluding chemicals outside the defined AD presents a limitation. In contrast, Viganò et al. (Viganò et al., 2024) developed AI models that use Adverse Outcome Pathways (AOP) to predict cardiotoxicity by connecting chemical structures to molecular initiating events (MIEs) and downstream key events (KEs). Their central natural language processing (NLP) model, trained on SMILES representations, achieved an ACC of 0.815, with performance improving as more training data were used. However, the resulting structure-based predictions remained less interpretable than mechanistically grounded approaches. Despite issues like data imbalance, the study offers a comprehensive toolkit aligned with New Approach Methodologies (NAMs) for early-stage chemical safety assessment. Furthermore, Seal et al. (Seal et al., 2024) developed the first publicly available ML models that used chemical and biological data to predict the FDA’s DICTrank. The best-performing model in that study used Mordred physicochemical descriptors and achieved a PR-AUC of 0.93; the analysis also identified structural alerts and biological processes associated with cardiotoxicity, including hERG inhibition. However, models based on more mechanistic biological data encountered limitations due to data scarcity. The study also provides a freely accessible web tool for rapid cardiotoxicity screening. Meanwhile, Yang et al. (Yang et al., 2025) found that large language models (LLMs) can effectively predict heart-related toxicity in molecules. Their study evaluated specialized GPT-4 models for specific cardiac targets. The GPT-4-CaV1.2 model achieved an ACC of 0.82 and was used to identify potentially cardiotoxic substances found in traditional Chinese treatments. However, a notable limitation is that, although the specialized GenAI model slightly outperformed the general-purpose GPT-4 model, their overall performance was broadly comparable, with differences that were not always statistically significant. Liu et al. (Liu K. et al., 2025) developed ML and DL models to predict the cardiotoxicity of medications by analyzing their interactions with the hERG channel. A transformer utilizing Morgan fingerprints achieved a ROC-AUC of 0.93. To enhance interpretability, SHAP analysis was used to identify key structural features that affect toxicity. However, a significant challenge remains in balancing the model’s simplicity with its explanatory power. In contrast, Ahmadi et al. (Ahmadi et al., 2025) developed interpretable ML models to predict chemotherapy-induced cardiotoxicity in breast cancer patients through the use of echocardiography radiomics. The KNN model, applied to the short-axis view, demonstrated strong performance, achieving an ACC of 0.92 and an ROC-AUC of 0.69. They used SHAP, along with a permutation-based feature importance test, to improve the model’s explanations. However, the study’s limited sample size and single-center design limit its generalizability. Talebi et al. (Talebi et al., 2025) developed an ML model to predict cardiotoxicity related to radiation therapy in breast cancer patients. An SVM combining dosimetric, clinical, and radiomic features achieved a ROC-AUC of 0.97. While adding radiomics improved the model’s predictive ability, it also increased its complexity, potentially affecting clinical interpretability. The study has notable limitations, including its focus on left-sided tumors and a small sample size. Concurrently, Qu et al. (Qu et al., 2025) evaluated the DICTrank dataset as a resource for developing mechanism-agnostic ML models. Their study found that XGBoost and LR produced the strongest results among the evaluated models, with a reported ROC-AUC of approximately 0.61. They employed SHAP analysis to improve interpretability. However, a primary constraint is that the dataset is derived from FDA labels, which may not accurately reflect preclinical cardiotoxicity for new medication candidates. Liang et al. (Liang et al., 2026) proposed a DL framework, CardiacTox, for the prediction of nine categories of compound-induced cardiotoxicity with multi-view molecular graphs, Morgan and MACCS fingerprints, and molecular descriptors. The model is based on GNNs and bidirectional gated recurrent units (GRUs), achieving a ROC-AUC of 0.938 for predicting CaV1.2 and the highest external validation ACC of 82.2% on the hERG-70 dataset. The authors used SHAP analysis, graph-attention visualization, and toxicity-alert substructure mining to explain the model and identify molecular features and structural patterns associated with cardiotoxic effects. As an offline, structure-based tool, CardiacTox does not take into account pharmacokinetic, clinical, or patient-specific factors, which may limit its application in real-time and personalized cardiotoxicity assessment. Table 4 summarizes the reviewed studies on cardiotoxicity. Recent studies in computational cardiotoxicity prediction have reported strong predictive performance for targets such as hERG. However, a significant challenge in this field is finding the right balance between model effectiveness and interpretability. Tools such as SHAP are often used to address this issue. Furthermore, the field faces concerns about generalizability due to small sample sizes, reliance on historical data, and limited applicability.

**TABLE 4:** Overview of previous research efforts in cardiotoxicity prediction.

Study	Dataset(s)/sample size (N)	Endpoint	Positive/Negative	Features	Training model(s)	Split/validation	Best performance	Independent validation	Major limitation(s)
Ye et al. (2022)	ChemIDplus, SIDER Pharmapendium Tox21	Drug-induced cardiotoxicity (DICT)	402/244	ECFP, ToxPrint, Tox21 assay features	SVM, RF, NB, XGBoost	70/30 train–validation split	NB (ROC-AUC 0.83)	Not reported	Limited chemical-space coverage
Delre et al. (2022)	ChEMBL (v25)	hERG-mediated cardiotoxicity	1,229/6734 (pIC50 = 6); 3847/4116 (pIC50 = 5)	DRAGON descriptors	RF, KNN, GB, XGBoost, SVM and MLP	80/20 + 5-fold cross-validation + temporal external validation	balanced random forest (BRF) + SVM (BA 0.91, ROC-AUC 0.95)	Temporal validation	Strict AD reduces chemical coverage
Viganò et al. (2024)	ICE	adverse outcome pathway (AOP)-related cardiotoxicity	184/48 (MIE1) 191/445 (KE1) 1,147/3857 (KE2)	Mordred descriptors, fingerprints (Morgan, MACCS), Graph, embedding	LR, DT, RF, BRF, NB, KNN, DNNs, MPNNs, NLP model, and Multimodal Neural Network	90/10 split (MIE1/KE1); 70/30–90/10 splits (KE2); 10× k-fold CV; 5-fold CV tuning	NLP (ACC 0.815)	Not reported	Class imbalance; limited sample size for MIE1/KE1; interpretability trade-off
Seal et al. (2024)	DICTrank + SIDER, Cell Painting, LINCS, DrugBank, *etc.*,	FDA DICTrank cardiotoxicity	742/278	Morgan fingerprints, Mordred, MOA, protein targets, imaging, transcriptomics	RF + ensembles	Cross-validation/held-out evaluation	RF (PR-AUC 0.93, ROC-AUC 0.84)	Not reported	Biological features affected by data scarcity
Yang et al. (2025)	Toxicogenomic, SIDER, FAERS, PubChem, DrugBank	Cardiac-target toxicity	441/361 (Cav1.2); 10,404/11,842 (hERG); 1,143/926 (Nav1.5)	SMILES, Graphs, Morgan fingerprints	WeaveGNN, Morgan fingerprints MLP, SVC, KNN, GPT-4, GPT-4o	80% development/20% held-out validation + 5-fold CV during training	GPT-4-CaV1.2 (ACC 0.82)	Yes	limited specialized biochemical knowledge of GPT-4
Liu et al. (2025b)	PubChem, ChEMBL, BindingDB, PubMed	hERG cardiotoxicity	Train: 7055/10,742; Test: 1775/2686	RDKit + Morgan, MACCS, AtomPairs, FP2	SVM, RF, KNN, XGBoost, MLP DNN, Transformer, Ensemble Models	80/20 + external independent validation	Transformer (ROC-AUC 0.93)	Yes	Interpretability; complexity trade-off
Ahmadi et al. (2025)	Cardio-Oncology Dept., Rajaei Center, Tehran; N = 100	Post-chemotherapy cardiotoxicity	41/59	184 2D features (texture, shape, frequency)	RUS Boosted, RF SVM, KNN, and NB	80/20 + 5-fold CV	KNN (ACC 0.92, ROC-AUC 0.69)	No	Small, single-center clinical cohort
Talebi et al. (2025)	Rajaei Center; N = 83 breast-cancer patients	Radiotherapy-induced cardiotoxicity	Not reported	Dosimetric, Demographic, Clinical, and Radiomic	SVM, RF, NN and NB	70/30+ internal 5-fold CV	SVM (ROC-AUC 0.97)	No	Small sample size; LV-focused cardiotoxicity
Qu et al. (2025)	DICTrank	Drug-induced cardiotoxicity	Train: 485/136 Test: 193/110	Mold2 descriptors	LR, KNN, SVM, RF, XGBoost	5-fold CV	XGBoost (ACC 0.64, ROC-AUC 0.60)	Not reported	FDA-label-derived endpoint may not represent preclinical toxicity
Liang et al. (2026)	Arrhythmia, Cardiac Failure, Cardiotoxicity, Heart Block, Hypertension, Myocardial, hERG, CaV1.2, and NaV1.5	Arrhythmia, heart failure, hERG, CaV1.2, NaV1.5*etc.*	706/806 (Arrhythmia); 307/846 (Cardiac Failure); 922/400 (Cardiotoxicity); 466/466 (Heart Block); 705/669 (Hypertension); 401/402 (Myocardial); 530/272 (CaV1.2); 1,414/655 (NaV1.5); 6637/5977 ()	Molecular graphs, RDKit descriptors Morgan, MACCS fingerprints	Multi-view GNN/graph attention	60/20/20 + 5-fold CV	CaV1.2 (ROC-AUC 0.938), hERG-70 (ROC-AUC 0.82)	Yes	lacks dynamic clinical/PK information, ECG signals, drug exposure, concomitant medications, and patient-specific factors

### 5.3 Nephrotoxicity

Shi et al. (Shi et al., 2022) developed a consensus model that achieved a ROC-AUC of 0.93 for predicting drug-induced nephrotoxicity. To improve interpretability, they identified and shared 87 structural alerts linked to kidney failure. The study illustrates the challenge of balancing predictive performance with mechanistic clarity. Nephrotoxicity is a complex endpoint that may not be adequately described by individual simple descriptors, and structural-alert methods have limited ability to represent spatial relationships among substructures. Gong et al. (Gong et al., 2022) developed ML models to predict drug-induced nephrotoxicity, with an SVM using the GraphFP representation, achieving a ROC-AUC of 0.915. Their framework, based on Organisation for Economic Co-operation and Development (OECD) guidelines, established AD and identified structural alerts. However, specificity exceeded sensitivity, and substantial structural similarity between nephrotoxic and non-nephrotoxic compounds complicated classification. In contrast, Mazumdar et al. (Mazumdar et al., 2023) evaluated a deep neural network (DNN) alongside 27 ML models to predict renal toxicity, with the Extra Trees (EXT) model achieving a test ROC-AUC of 0.87. The study also applied association-rule mining to identify 10 combination-based structural alerts that capture relationships beyond individual substructures. The study highlights the dependence of model performance on data availability and molecular representation. Additionally, the identified structural alerts require wet-lab validation to confirm their association with renal toxicity. Similarly, Rao et al. (Rao et al., 2024) developed an ensemble AI model that integrated physicochemical features with off-target interaction data to predict drug-induced kidney injury. The ensemble model achieved a ROC-AUC of 0.87. Their combined approach outperformed models that relied on a single data type; however, the research faced limitations due to a small dataset and the potential risk of overfitting. Additionally, Chenga et al. (Chenga et al., 2025) reported higher predictive performance for models trained on transcriptomic data than for models trained on chemical descriptors in their evaluation. Their gene-expression-based LightGBM model with SPXY sampling (GEM20) achieved the highest reported ROC-AUC of 0.94. They employed SHAP analysis to pinpoint critical genes and improve interpretability. However, transcriptomic modeling can be less practical because gene-expression data are more costly and difficult to obtain than structure-only inputs. Additional limitations include the inherent class imbalance in transcriptomic data, which requires specialized methods, such as the GHOST algorithm, to determine optimal prediction thresholds. Banerjee and Roy (Banerjee and Roy, 2025) developed ML-based c-RASAR models to predict the nephrotoxicity of orally active drugs using 0–2D molecular descriptors and MACCS fingerprints. A total of 36 QSAR and c-RASAR models were developed using 9 ML algorithms and evaluated using 20-times repeated fivefold cross-validation. Based on the Sum of Ranking Differences (SRD) multi-criteria analysis, the descriptor-based LDA c-RASAR model was selected as the best overall model, achieving an ACC of 0.718, BA of 0.714, and ROC-AUC of 0.710 on the test set. The model was further evaluated on a true external set of 111 nephrotoxic DrugBank compounds, correctly identifying 73 compounds with a sensitivity of 0.658. ARKA analysis was additionally employed to identify activity cliffs and improve interpretation of the chemical space. However, the relatively small modeling dataset of 317 drugs and the use of an external validation set containing only nephrotoxic compounds limit comprehensive assessment of external specificity and overall classification performance. Finally, Amin et al. (Amin et al., 2026) presented KidneyTox_v1.0, an ML model for predicting drug nephrotoxicity based on the molecular descriptors of 565 compounds. The optimized RF classifier achieved an ACC of 84.1% after optimization with the Optuna algorithm. SHAP values were used to identify the most influential predictive features, while applicability domain analysis was performed to evaluate their predictive confidence. Key limitations are the modest dataset size and the need for external validation using larger and more diverse datasets. Table 5 summarizes the reviewed studies on nephrotoxicity. Recent advances in nephrotoxicity prediction modeling leverage integrated data and ensemble algorithms to improve accuracy. XAI methods support interpretation by identifying molecular features associated with model predictions. A recurring objective is to balance predictive performance with interpretable outputs that can support drug-discovery applications. Nonetheless, progress in this area faces obstacles related to data acquisition, experimental validation, and the inherent trade-off between complexity and interpretability associated with advanced computational methods.

**TABLE 5:** Overview of previous research efforts in nephrotoxicity prediction.

Study	Dataset(s)/sample size (N)	Endpoint	Positive/Negative	Features	Training model(s)	Split/validation	Best performance	Independent validation	Major limitation(s)
Shi et al. (2022)	SIDER, Zhang et al.; N = 565	Drug-induced nephrotoxicity/kidney failure	Train: 232/224 Validation: 55/54	Descriptors (Chemaxon, Fragmentor, GSFrag, MORDRED, alvaDesc, QNPR, PyDescriptor, RDK)	ASNN, SVM, DT, XGBoost, RF, CNF, TRANSNN, TEXTCNN, GIN, EAGCNG and consensus	5-fold CV + external validation	Consensus external-validation ACC: ACC/Q 0.86, ROC-AUC 0.93	Yes	Complex endpoint not fully captured by simple descriptors
Gong et al. (2022)	SIDER, DrugBank, ChEMBL, TCM@taiwan, FDA, … ; N = 777	Drug-induced nephrotoxicity	Train: 303/396	Atom Pair 2D, E-state, CDK, MACCS, Klekota-Roth, PubChem, Substructure and related fingerprints	ANN, LightGBM RF, SVM, NB, DT, KNN, and XGBoost	90/10 + 10-fold CV + external validation	SVM_GraphFP (ROC-AUC 0.915, ACC 0.857)	Yes	Specificity exceeded sensitivity; some nephrotoxic and non-nephrotoxic compounds had highly similar structures
Mazumdaret al. (2023)	Shi et al.; N = 565	Drug-induced renal toxicity	287/278	RDKit descriptors; MACCS, Extended, Graph-only, Klekota-Roth, Atom Pair, Substructure, E-State, PubChem fingerprints	DNN, NB XGBoost EXT, LR, SVM, KNN, RF, CNN, Transfer Learning	80/20 +5-fold CV on training set	EXT (ROC-AUC 0.87 ACC 0.82)	No	Limited data availability; sensitivity to data representation
Rao et al. (2024)	FDA, DIRIL; N = 360	Drug-induced kidney injury	231/129	Physicochemical Properties, Gene Interaction (Off-target) Indicators	RLR, SVM, RF, NN, ensemble	70/30 + 5-fold CV for tuning	Ensemble (ROC-AUC 0.87, ACC 0.79)	No	Small dataset
Chenga et al. (2025)	Gong et al., Shi et al., Kwiatkowska et al., Patel et al., LINCS L1000	Binary drug-induced nephrotoxicity	Chemical: 252/168; transcriptomic profiles: 6,162/18,825	PaDEL descriptors, fingerprints (CDK, Extended CDK Estate, MACCS, GraphOnly, PubChem, Pair2D, Atom, and Substructure)	LightGBM, SVM RF, and XGBoost	75:25 +5-fold CV + SR, SPXY and sphere-exclusion splitting	GEM20 (LightGBM): ROC-AUC 0.94, ACC 0.87	No	Transcriptomic data are costly, noisy and computationally demanding
Banerjee and Roy (2025)	Connor et al.; N = 317	Binary nephrotoxicity prediction	Not reported	0–2D descriptors, MACCS fingerprints	LDA, SVM, RF, LR, QDA, MLP, NB, GB, AdaBoost	5-fold CV	LDA c-RASAR: ACC 0.718; BA 0.714; Precision 0.73	Yes	Small dataset; external set contained only nephrotoxic drugs
Amin et al. (2026)	Shi et al., Toropov et al.; N = 565	Binary drug-induced nephrotoxicity	287/278	Mordred descriptors	RFC, qRASAR	80:20	RFC (ROC-AUC 0.84)	No	Small dataset; external validation with larger datasets needed

### 5.4 Hepatotoxicity

Jaganathan et al. (Jaganathan et al., 2021) developed an effective QSAR-SVM model for predicting DILI by selecting 155 molecular descriptors from a dataset of 1,253 compounds. The model achieved an internal 10-fold cross-validation ACC of 0.811. Additionally, they employed SHAP analysis to improve interpretability by identifying key predictive features. However, a significant limitation of the model is its exclusive reliance on structural data, which does not account for biological or dosage information. Meanwhile, Chen et al. (Chen et al., 2022) developed a ResNet18 DNN model to predict DILI using features extracted directly from molecular structure images. The model achieved a test ROC-AUC of 0.958 and ACC of 0.976; the corresponding training values were ROC-AUC 0.973 and ACC 0.992. However, because the model relies on molecular structure-image embeddings, its predictions are less directly interpretable in terms of the specific structural or physicochemical features driving DILI classification. Following this, Mostafa et al. (Mostafa et al., 2024) developed ML models to predict DILI using a large, clinically annotated dataset from NIH LiverTox. Their RF model reported a mean ACC of 63.1% and a recall of 71.6% and identified 90.9% of drug candidates in their analysis that had failed clinical trials because of hepatotoxicity. To enhance model interpretability, they employed SHAP analysis. However, the study also recognizes the challenges related to class imbalance in DILI datasets and the risk of overfitting associated with complex models. Khan et al. (Khan et al., 2024) proposed a hybrid ensemble model integrating ML and DL methods for hepatotoxicity prediction using a large dataset of 2,588 chemicals and drugs. The best voting ensemble model based on Morgan fingerprints achieved an ACC of 0.803, a ROC-AUC of 0.828, and a recall of 0.930. The predictive reliability of the model was further supported by 10-fold cross-validation and a held-out test set. However, the approach primarily relies on molecular descriptors and fingerprints and therefore does not incorporate biological, mechanistic, or exposure-related information that may influence hepatotoxicity. Similarly, Lee and Yoo (2024) developed InterDILI, an interpretable ML framework for DILI prediction; the RF model achieved a ROC-AUC of 0.97. The framework combines global feature analysis with an attention mechanism to provide complementary levels of interpretation. This approach offers both comprehensive insights and detailed information for individual compounds. However, a notable limitation is that dose-dependent DILI was not considered, despite dosage being an important factor influencing hepatotoxicity. Furthermore, the molecular fingerprint hashing procedure may map multiple substructures to the same feature, introducing ambiguity in structural interpretation. In contrast, Wang et al. (Wang et al., 2024) introduced DMFPGA, a DL model with a ROC-AUC of 0.935, designed to predict DILI by integrating both graph and fingerprint features. The combination of graph and fingerprint features improved predictive performance in the reported experiments, although the model’s complexity limits interpretability. Additional limitations include the need to evaluate a broader range of molecular properties and optimize hyperparameters. Amin et al. (Amin et al., 2025) developed a comprehensive framework for predicting DILI by integrating structural alerts with a high-performance RF model. This approach achieved an ACC of 0.77 and an F1-score of 0.84. To enhance interpretability, they employed Bayesian toxicophore detection and partial dependence plots to highlight key molecular features. The user-friendly tool, pDILI_v1, is designed for early screening. However, a notable limitation is that it does not account for clinical factors, such as dosage and patient genetics. In addition, Lee and Posma (2025) developed DILIGeNN, a graph neural network framework for predicting drug-induced liver injury (DILI) using custom molecular graph representations derived from 3D-optimized molecular structures. The framework incorporates structural and electrostatic features, including bond lengths, Gasteiger partial charges, and total and explicit valence. Several GNN architectures, including GCN, GAT, GIN, and GraphSAGE, were evaluated using nested cross-validation. The best-performing GraphSAGE model achieved a ROC-AUC of 0.897, an ACC of 0.836, an F1-score of 0.846, and an MCC of 0.698 on the DILI test sets. However, the model relies exclusively on chemical features without incorporating biological information, and the study did not focus on explaining the model’s decision-making process, limiting mechanistic interpretability. Furthermore, the force-field-derived 3D features may not fully represent molecular conformations during biological interactions. Duong et al. (Duong et al., 2026) proposed a DL algorithm to predict DILI using a benchmark dataset containing 475 molecules in common molecular images and two heatmap representations. The best-performing ResNet-34/HM2 configuration achieved a ROC-AUC of 0.88. Functional-group analysis and Grad-CAM were used to improve model interpretation. However, the small size of the dataset and reliance on structural information may limit the model’s generalizability. Finally, Liu et al. (Liu et al., 2026) introduced a DL framework named MolFPKG-DILI, based on attention-based feature fusion and relational graph convolutional networks. The framework integrates molecular fingerprints, molecular graph features, and a drug-centered knowledge graph to predict drug-induced liver injury (DILI) and assess toxicity severity. The model achieved an ACC of 72.73% for DILI/No-DILI classification, 75.81% for Most-DILI/Less-DILI classification, and 82.35% on an independent validation set of 17 drugs for DILI prediction. SHAP, saliency analysis, and knowledge-graph interpretation were used to identify important molecular and biological features associated with the predictions. However, the model does not explicitly incorporate dose–response relationships or pharmacokinetic information. In addition, its static knowledge graph requires retraining when new drugs or information are introduced, and severity prediction is limited to only two levels, reducing the granularity of DILI severity assessment. Table 6 summarizes the reviewed studies on hepatotoxicity. Recent advances in predicting hepatotoxicity illustrate the value of integrating ensemble methods with advanced DL architectures to improve prediction accuracy. Researchers are developing XAI frameworks that leverage SHAP, attention mechanisms, and structural alerts to enhance understanding. These studies aim to achieve a balance between high predictive performance and mechanistic interpretability. However, the lack of standardization in data and pharmacological variables, such as dosage, hinders progress.

**TABLE 6:** Overview of previous research efforts in hepatotoxicity prediction.

Study	Dataset(s)/sample size (N)	Endpoint	Positive/Negative	Features	Training model(s)	Split/validation	Best performance	Independent validation	Major limitation(s)
Jaganathanet al. (2021)	DILIrank, LiverTox, LTKB, PubMed N = 1,253	Binary DILI	Train: 636/617; external: 94/114	Descriptors (RDKit, PaDEL, Chemopy, CDK)	SVM, MLP, LR, RF, XGBoost, KNN, NB, DT	10-fold CV	SVM Internal 10-fold CV (ACC 0.811, sensitivity 0.840)	Yes	Structure-descriptor dependence; no biological or dose information
Chen et al. (2022)	DILIrank, LiverTox, Hepatox, SIDER, LTKB, … ; N = 1,446	hepatotoxicity prediction	Train: 1,060/239 Test: 129/18	Molecular Structure Images (2D)	ResNet18DNN	90/10	Test (ROC-AUC 0.958, ACC 0.976, recall 0.935) Train (ROC-AUC 0.973, ACC 0.992)	No	class imbalance; dependence on molecular image
Mostafa et al. (2024)	NIH LiverTox DILI likelihood dataset N = 575	Binary DILI likelihood	240/335	1D and 2D Mold2 descriptors	RF, SVM, DT, KNN, MLP	80/20 + 10-fold CV	internal CV RF (ACC 0.63 Recall 0.71)	Yes	Limited availability; class imbalance
Khan et al. (2024)	CTD, Haz Map/NLM Xu et al., Greene et al., N = 2588	Binary hepatotoxicity	1,618/970	RDKit molecular descriptors, Mordred descriptors, Morgan fingerprints	SVC, KNN, RF, ET, RNN, MLP	80/20 + 10-fold CV	Voting Ensemble Model IV + Morgan fingerprints (ACC 0.8026, ROC-AUC 0.8284)	Yes	limited data availability; slight class imbalance; risk of overfitting
Lee and Yoo (2024)	NCTR, Greene, Xu, Liew, DILIrank	Binary DILI	Train: 768/630 Test: 184/268	ECFP6 FP, 8 physicochemical descriptors	RF, Light GBM, LR, and NN with attention	Source-based hold-out validation + repeated stratified 10-fold CV	RF on independent (ACC 0.90, sensitivity 0.96)	Yes	ECFP6 may miss molecular substructures larger than radius 3
Wang et al. (2024)	LiverTox, LTKB, Hepatox, DILIst, DILIrank, and literature-derived data; N = 1,888	Binary DILI prediction	1,033/855	Graph, Fingerprints (ECFP, MACCS, ErG)	DMFPGA, RF, GCN, MPNN, ResNet18	80/20+5-fold CV	DMFPGA (ACC 0.934, ROC-AUC 0.935)	No	Limited fingerprint/feature selection space and substantial hyperparameter optimization requirements
Amin et al. (2025)	Publicly available DILI dataset; N = 1,159	hepatotoxicity prediction	Train: 572/355 Test 145/87	Mordred descriptors, ECFP-6 FP	LR, KNN, NB RF, DT, QDA, MLP	80/20 diversity-based train/test split+ 5-fold CV + StratifiedKFold tuning	RF (ACC 0.77, F1-score 0.83)	No	Does not account for clinical factors such as dosage and patient genetics
Lee and Posma (2025)	FDA DILIst; N = 1,167	Binary DILI prediction	768/511	3D-optimized molecular graphs	GCN, GAT, GIN, GraphSAGE	Nested 4-fold outer CV with 5-fold inner CV for hyperparameter tuning	GraphSAGE: (ROC-AUC 0.897; ACC 0.836; MCC 0.698)	No	optimized 3D features may not reflect true biological conformations
Duong et al. (2026)	FDA/NCTR DILI benchmark dataset; N = 475	hepatotoxicity prediction	236/239	NM + Crippen-logP HM1 + logBCF-based HM2 molecular images	ResNet-18/34/50; EfficientNet-B0–B3	Stratified 4-fold CV	ResNet-34 with HM2 (ROC-AUC 0.88)	No	Small dataset; pharmacokinetic and metabolic information not incorporated
Liu et al. (2026)	DILIrank + DILIst N = 824	DILI vs. No-DILI; Most-DILI vs. Less-DILI	DILI: 420/404; Severity: 153/153	MACCS, Morgan, ErG, molecular graph, knowledge graph	MolFPKG-DILI FPNN + GCN + attention + RGCN	5-fold CV	ACC 0.73 (DILI); ACC 0.76 (severity)	Yes	limited severity levels; no dose-response/PK data

### 5.5 Neurotoxicity

Banerjee et al. (Banerjee et al., 2024) enhanced the ProTox 3.0 platform by integrating a specialized RF neurotoxicity model. This model achieved a BA of 86% and a ROC-AUC of 0.87 during validation. It uses molecular fingerprints for prediction; however, it lacks built-in structural-alert visualization, which limits direct interpretation of its predictions. The model was also applied to compounds such as methandrostenolone, illustrating its potential utility for screening. Concurrently, Gao et al. (Gao et al., 2025) developed an ML model to predict neurotoxicity based on molecular structure, achieving an ACC of 86.56% with an SVM using Morgan fingerprints. They utilized SHAP analysis to identify and explain the key substructures of neurotoxic chemicals, thereby enhancing the model’s interpretability. However, a significant limitation of the model is its primary focus on molecular properties, which neglects important biological, genetic, and systemic interactions that may influence neurotoxicity. Similarly, Hemmdan et al. (Hemmdan, 2025) developed a DL model to predict acetylcholinesterase (AChE)-inhibition activity, achieving an ACC of 85.25% and a ROC-AUC of 0.91. Principal component analysis (PCA) was used for dimensionality reduction; however, AChE inhibition is a surrogate endpoint and does not constitute direct assessment of neurotoxicity. In contrast, Hao et al. (Hao et al., 2025) developed an XGBoost model for the binary classification of neurotoxicity using a dataset comprising 2,670 chemicals with a training ACC of 0.93. SHAP analysis was incorporated to identify molecular features that contributed to the predictions. The model demonstrated practical applicability by screening 1,145 human blood molecules and identifying 36 as high risk. However, a notable limitation is that it can only perform binary classification for organic chemicals and does not estimate neurotoxicity severity. Further, Pang et al. (Pang et al., 2025) developed NeuTox 2.0, a hybrid DL model to predict four neurotoxicity endpoints. Their neuronal-cytotoxicity model achieved a ROC-AUC of 0.964. To enhance the model’s interpretability, they used SHAP analysis. When applied to the Registration, Evaluation, Authorisation and Restriction of Chemicals (REACH) database, the model identified 701 compounds of potential concern, illustrating its possible utility for initial screening. However, model performance varied across neurotoxicity endpoints, reflecting differences in dataset characteristics and experimental outcomes, while limited labeled data and the relatively simple feature-fusion strategy remain important challenges. Finally, Jiang et al. (Jiang et al., 2026) proposed an ML model for brain-specific neurotoxicity prediction based on traditional molecular fingerprints, KPGT graph embedding, and MolFormer-based molecular representation. Among the evaluated models, KPGT-MLP achieved an ACC of 89.28% and a ROC-AUC of 0.94. SHAP analysis was applied to highlight molecular features associated with the neurotoxicity predictions. However, important limitations include the lack of mechanistic biological information and *in vivo* validation, as well as the need for validation on fully independent external datasets. Table 7 summarizes the reviewed studies on neurotoxicity. Recent neurotoxicity studies use a range of ML and DL approaches, including tree-based models and neural architectures, across diverse chemical datasets. A notable advancement in this field is the integration of XAI, particularly SHAP, which can help identify influential structural features while improving model transparency. These techniques are valuable for analyzing extensive chemical inventories, such as those in REACH and human blood, to identify the most relevant substances for further investigation. Nevertheless, challenges remain, including the binary nature of predictions, the exclusion of inorganic substances, and the ongoing trade-off between model complexity and full interpretability.

**TABLE 7:** Overview of previous research efforts in neurotoxicity prediction.

Study	Dataset(s)/sample size (N)	Endpoint	Positive/Negative	Features	Training model(s)	Split/validation	Best performance	Independent validation	Major limitation(s)
Banerjee et al. (2024)	Giorgini/Banerjee/Zhao-related neurotoxicity dataset	Binary drug-induced neurotoxicity	Not reported	Fingerprints (MACCS, Morgan)	RF	10-fold CV + Randomized SearchCV + external validation	ACC 0.86 ROC-AUC 0.87	Yes	Limited visualization of structural alerts
Gao et al. (2025)	ChEMBL	Binary neurotoxicity classification	1,622/1,499	RDKit descriptors, Fingerprints (Morgan, RDKit, MACCS, Topological Torsion)	SVM, RF, XGBoost MLP, GNN, WeaveGNN	80/20	Morgan-fingerprint SVM (ACC 0.86, ROC-AUC 0.93)	Yes	biological, genetic, and environmental factors are not incorporated
Hemmdan (2025)	ChEMBL; N = 4,695 molecules initially collected	AChE inhibition as a neurotoxicity surrogate	1848/1808	PaDEL descriptors and fingerprints (E-state, MACCS, PubChem, KlekotaRoth, AtomPairs 2D Substructure, CDK fingerprints, CDK extended, CDK graph only)	Seven-layer NN	Train/test split; exact ratio not reported	ACC 0.85, ROC-AUC 0.91	No	AChE inhibition used as a surrogate for neurotoxicity
Hao et al. (2025)	U.S. Environmental Protection Agency (EPA) CompTox, SIDER; N = 2,670	Binary neurotoxicity	Train: 1,405/720; Validation: 183/90 Test: 181/91	Molecular graphs, 7 physicochemical descriptors, PubChem fingerprints	GAT, RF, DT, SVM, LR, KNN Transformers, and XGBoost	8:1:1+ 5-fold CV	XGBoost: 5-fold CV (ROC-AUC 0.87); held-out test (ROC-AUC 0.75); train (ACC 0.93)	Yes	Excludes high-molecular-weight, inorganic, organometallic compounds, and mixtures
Pang et al. (2025)	BBB N = 12,901 NC N = 2,024 NA N = 1,018; NT N = 2,612	blood-brain barrier (BBB) permeability, SH-SY5Y neuronal cytotoxicity, MEA-based	BBB 8,039/4,862; NC 902/1,122; NA 316/702; NT 1,769/596	PaDEL descriptors, ECFPs D-MPNN, MFBERT	LightGBM, NeuTox 2.0 - A hybrid deep learning model	8:1:1	NeuTox 2.0 test results: BBB: ACC 0.91; NC: ACC 0.91, NA: ACC 0.80; NT: ACC 0.79, ROC-AUC 0.83	Yes	Limited labeled data; performance variability across endpoints
Jiang et al. (2026)	CTD, PubChem; N = 2,144	Binary brain-specific neurotoxicity classification	948/1,196	KPGT graph embeddings MolFormer transformer embeddings, SMILES-BERT, Fingerprints (Morgan, RDKFP)	RF, XGB, MLP, SVM, GNN	Stratified 80:20+ 5-fold CV	KPGT-MLP (ACC 0.89, ROC-AUC 0.94)	No	Lack of mechanistic and *in vivo* validation

### 5.6 Cross-organ toxicity comparison

The five organ-specific toxicity domains differ significantly in dataset size, endpoint definition, molecular representation, class distribution, and validation design. Larger datasets were reported in several neurotoxicity, hepatotoxicity, and respiratory-toxicity studies. For example, Hao et al. used 2,670 chemicals for neurotoxicity prediction (Hao et al., 2025), Khan et al. used 2,588 chemicals and drugs for hepatotoxicity prediction (Khan et al., 2024), and Wang et al. used 2,529 compounds for respiratory-toxicity prediction (Wang et al., 2021). In contrast, KidneyTox_v1.0 included only 565 compounds (Amin et al., 2026), while the cardiotoxicity study by Talebi et al. involved only 83 breast-cancer patients (Talebi et al., 2025). Such differences in dataset size and composition can substantially influence model robustness and generalizability. Therefore, reported performance should be interpreted in the context of the quantity, diversity, and quality of the available data rather than headline performance metrics alone. The corresponding dataset sizes are reported in the organ-specific tables. The biological relevance of the prediction tasks also varies considerably across organ systems. Hepatotoxicity studies commonly focus on DILI (Jaganathan et al., 2021; Chen et al., 2022; Mostafa et al., 2024; Wang et al., 2024), whereas cardiotoxicity studies may target specific molecular mechanisms or targets such as hERG and CaV1.2 (Liu K. et al., 2025; Liang et al., 2026). Neurotoxicity studies encompass more heterogeneous endpoints, including blood–brain barrier permeability, neuronal cytotoxicity, neural activity, mammalian neurotoxicity, and drug-induced neurotoxicity (Pang et al., 2025). Nephrotoxicity studies commonly classify compounds according to drug-induced nephrotoxicity or renal-toxicity outcomes (Shi et al., 2022; Gong et al., 2022; Duong et al., 2026), whereas respiratory-toxicity studies use respiratory or pulmonary toxicity outcomes as prediction endpoints (Wang et al., 2021; Jaganathan et al., 2022; Jaganathan et al., 2025). Because these biological endpoints, dataset characteristics, and prediction tasks are not directly equivalent, a higher ROC-AUC or ACC in one organ domain does not necessarily indicate a superior modeling approach. Validation rigor also differs substantially across studies. For respiratory-toxicity prediction, Jaganathan et al. used 1,869 training compounds, 465 test compounds, and an independent external validation set of 193 compounds (Jaganathan et al., 2025). In hepatotoxicity prediction, Jaganathan et al. developed the model using 1,253 compounds, comprising 636 hepatotoxic and 617 non-hepatotoxic compounds, and subsequently evaluated it using an independent external set comprising 94 positive and 114 negative compounds (Jaganathan et al., 2021). Some nephrotoxicity and cardiotoxicity studies also employed independent external validation (Shi et al., 2022; Gong et al., 2022), whereas other studies relied primarily on k-fold cross-validation or held-out test sets (Viganò et al., 2024; Seal et al., 2024; Yang et al., 2025; Hao et al., 2025; Khan et al., 2026). Consequently, strong performance obtained through internal cross-validation or a hold-out subset of the original dataset should be interpreted cautiously when independent external validation is absent.

Differences in molecular representation further complicate direct comparisons across organ systems. Traditional molecular descriptors and fingerprints, including Morgan, MACCS, PubChem, ECFP, and physicochemical descriptors, have been widely used in the reviewed studies (Seal et al., 2024; Liang et al., 2026; Wang et al., 2024; Amin et al., 2025; Banerjee et al., 2024; Gao et al., 2025), whereas more recent approaches use molecular graphs, pre-trained molecular embeddings, transformers, and multimodal feature-fusion strategies (Chushak et al., 2025; Liu K. et al., 2025; Pang et al., 2025). Consequently, the performance of a particular architecture depends not only on the learning algorithm but also on dataset size, endpoint complexity, and the molecular information encoded by the selected representation. Conventional ML approaches such as RF, SVM, XGBoost, and LightGBM may remain competitive on smaller datasets, whereas DL, graph-based, and multimodal approaches may provide additional advantages when larger and more diverse datasets or richer molecular representations are available (Guo et al., 2023; Seal et al., 2025). Overall, the available evidence indicates that model reliability across organ-specific toxicity domains is not determined by any single ML or DL architecture. Rather, it reflects the interaction among data quality and size, endpoint definition, molecular representation, class balance, and validation rigor. Therefore, direct comparison of reported ROC-AUC, ACC, or other performance measures across organ systems should be made cautiously and with careful consideration of differences in dataset composition and evaluation methodology (Guo et al., 2023).

## 6 Limitations and future directions

AI has shown considerable promise in toxicity prediction; however, its clinical and regulatory utility remains limited by data quality, small and imbalanced datasets, inconsistent endpoint definitions, uncertain negative labels, and differences in molecular representation and validation strategy. Models that perform well on internal datasets or random train/test splits may not generalize to structurally or biologically different external chemical spaces. Therefore, more rigorous validation using scaffold-based, temporal, prospective, and independent external datasets is important for assessing real-world generalizability (Seal et al., 2025). Reproducibility and standardization are also major concerns. Differences in data preprocessing, molecular standardization, dataset versions, benchmark splits, hyperparameters, assay conditions, and evaluation metrics can hinder meaningful comparison across studies. Greater transparency in source code, preprocessing pipelines, datasets, model parameters, and reporting practices is therefore needed to improve reproducibility and cross-study comparability (Schür et al., 2025). Future work should also address uncertainty quantification and domain-shift assessment (Parrondo-Pizarro et al., 2026), while active learning and federated learning may help overcome limited labeled data and support collaborative model development (Gangwal et al., 2024). Foundation models and pre-trained molecular models may strengthen molecular representation learning (Delile et al., 2025), whereas multimodal learning can combine complementary molecular representations, including molecular graphs, SMILES strings, structural images, and spectroscopic information (Park and Lee, 2025). Improved negative-data construction, assay and endpoint harmonization, causal and mechanistic modeling, integration of biological evidence, and prospective validation will also be important for developing AI-based toxicity models that are more reliable, interpretable, generalizable, and suitable for real-world regulatory applications.

## 7 Conclusion

This review discusses the growing role of artificial intelligence (AI) in predictive toxicology, emphasizing its potential to support earlier and more informative assessments of chemical and drug safety across multiple organ-specific toxicity domains. AI-based models have demonstrated competitive and, in some studies, improved predictive performance compared with traditional rule-based and simpler descriptor-based approaches. However, their relative performance depends strongly on dataset quality and size, endpoint definition, molecular representation, class distribution, validation strategy, and the specific baseline model used for comparison. Therefore, AI and DL approaches should not be considered inherently superior to conventional methods. The review also highlights key toxicity databases and computational tools that support model development and evaluation. Despite recent progress, important challenges remain in the clinical and regulatory application of AI-based toxicity prediction, including data quality, class imbalance, model interpretability, limited chemical-space coverage, applicability-domain constraints, reproducibility, uncertainty, and the need for robust independent external validation. Addressing these challenges through improved data standardization, transparent model development, rigorous validation, and careful use of explainable artificial intelligence (XAI) will be essential for improving the reliability and practical value of AI in drug safety. Continued collaboration among computational, experimental, clinical, and regulatory communities will be important for advancing the responsible application of these approaches.
